# Marine Bromophenol Bis (2,3-Dibromo-4,5-dihydroxy-phenyl)-methane Inhibits the Proliferation, Migration, and Invasion of Hepatocellular Carcinoma Cells via Modulating β1-Integrin/FAK Signaling

**DOI:** 10.3390/md13021010

**Published:** 2015-02-13

**Authors:** Ning Wu, Jiao Luo, Bo Jiang, Lijun Wang, Shuaiyu Wang, Changhui Wang, Changqing Fu, Jian Li, Dayong Shi

**Affiliations:** 1 Institute of Oceanology, Chinese Academy of Sciences, Qingdao 266071, China; E-Mails: wuning@qdio.ac.cn (N.W.); luojiao2012@163.com (J.L.); beckyjiang0220@163.com (B.J.); wanglijun@qdio.ac.cn (L.W.); 12-12sy@163.com (S.W.); 2 Qingdao Medical University Affiliated Hospital, Qingdao 266070, China; E-Mails: docjack@163.com (C.W.); changqing010336@163.com (C.F.); lijian_19891114@126.com (J.L.)

**Keywords:** bis (2,3-dibromo-4,5-dihydroxy-phenyl)-methane (BDDPM), anti-metastatic activity, cell adhesion, β1-integrin, FAK, BEL-7402 cell

## Abstract

Bis (2,3-dibromo-4,5-dihydroxy-phenyl)-methane (BDDPM) is a natural bromophenol compound derived from marine algae. Previous reports have shown that BDDPM possesses antimicrobial activity. In the present study, we found that BDDPM has cytotoxic activity on a wide range of tumor cells, including BEL-7402 cells (IC_50_ = 8.7 μg/mL). Further studies have shown that prior to the onset of apoptosis, the BDDPM induces BEL-7402 cell detachment by decreasing the adherence of cells to the extracellular matrix (ECM). Detachment experiments have shown that the treatment of BEL-7402 cells with low concentrations of BDDPM (5.0 μg/mL) significantly inhibits cell adhesion to fibronectin and collagen IV as well as cell migration and invasion. High doses of BDDPM (10.0 μg/mL) completely inhibit the migration of BEL-7402 cells, and the expression level of MMPs (MMP-2 and MMP-9) is significantly decreased. Moreover, the expression of β1-integrin and focal adhesion kinase (FAK) is found to be down-regulated by BDDPM. This study suggests that BDDPM has a potential to be developed as a novel anticancer therapeutic agent due to its anti-metastatic activity and also indicates that BDDPM, which has a unique chemical structure, could serve as a lead compound for rational drug design and for future development of anticancer agents.

## 1. Introduction

Bromophenol compounds are frequently isolated from various marine red algae and have been reported to exhibit a wide spectrum of pharmacological activities including antibacterial, antimicrobial, and antitumor activities [1,2,3,4]. Due to their multiple bioactivities, bromophenol compounds, which usually exist in marine sponges and algae, have attracted much attention from the researchers in the field of functional food and pharmaceutical agents. Previous studies have reported that some marine bromophenols, together with their derivatives, can inhibit the proliferation of many types of cancer cell lines *in vitro* [3,5,6,7]. Bromophenols isolated form red algae, as well as some synthesized isomers, have been reported to be cytotoxic against k562 cell lines [2]. The *Leathesia nana* extract containing large amounts of bromophenol derivatives inhibited the growth of Sarcoma 180 tumors in mice [7]. Accumulated evidence, both *in vitro* and *in vivo*, indicates that marine bromophenols may be a promising group of anticancer compounds.

**Figure 1:**
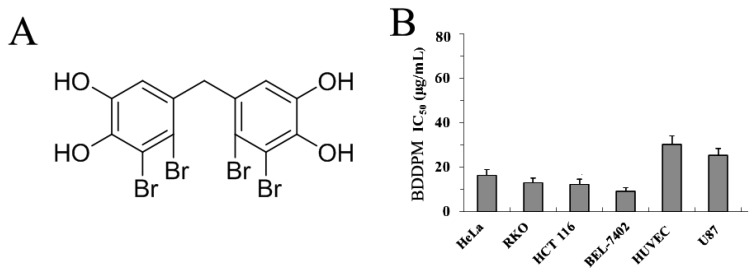
The structure of Bis-(2,3-dibromo-4,5-dihydroxy-phenyl)-methane (BDDPM) and its cytotoxic activity in cancer cell lines. Human cervical cancer cells (Hela), human colon cancer cells (RKO and HCT-116), human hepatoma cells (BEL-7402), and human vascular endothelial cells (HUVEC), as well as human glioblastoma cells (U87) were incubated in the absence or presence of certain concentrations of BDDPM for 24 h at 37 °C. MTT assay was performed to determine the growth inhibition of different cancer cells and HUVEC cells by BDDPM. The experiments were performed more than three times.

Bis (2,3-dibromo-4,5-dihydroxy-phenyl)-methane (BDDPM, Figure 1A), isolated from marine algae *L. nana* and *Rhodomela confervoides*, possesses various bioactivities, such as antimicrobial and antifungal activities [8]. We recently isolated and synthesized Bis (2,3-dibromo-4,5-dihydroxy-phenyl)-methane and found that it had PTP1B-inhibiting activity [9]. In the present research, we found that, among natural bromophenols, BDDPM displayed the highest anti-tumor activity against several cancer cell lines based on the 3-(4,5-dimethylthiazol-2-yl)-2,5-diphenyltetrazolium bromide (MTT) assay. However, the cytotoxic activity and the related molecular mechanism remain elusive.

The interaction between cells and extracellular matrix (ECM) plays a crucial role in cancer initiation and progression. The integrin family of cell adhesion receptors is the major mediator of cell adhesion to ECM, which links ECM to actin cytoskeleton at cellular structures called focal adhesions (or focal contacts) [10,11,12]. Besides integrins themselves, multiple structural and signaling molecules have been identified to be associated with focal adhesions, which highlight the importance of focal adhesions in the regulation of cellular structure and functions. Focal adhesion kinase (FAK), a non-receptor tyrosine kinase, is the first identified signaling molecule in focal adhesions. FAK-associated cell signaling plays an important role in cell motility and invasion. Integrin/FAK signaling has been shown to activate many signaling pathways through phosphorylation and protein-protein interactions to promote tumorigenesis [13,14,15,16]. FAK also plays a critical role in tumor progression and metastasis through its regulation of cancer cell migration, invasion, epithelial to mesenchymal transition, and angiogenesis, which are involved with both cancer cells and their microenvironment [17,18,19]. Here, we found that BDDPM could disturb the Integrin-FAK signaling, detach hepatocellular carcinoma cells from ECM, and abrogate their motility and invasiveness. BDDPM will be a potential novel Integrin-FAK inhibitor.

## 2. Results

### 2.1. BDDPM Inhibits Cancer Cell Proliferation

MTT assays were performed to investigate the effects of BDDPM on the proliferation of six cell lines. As shown in Figure 1B, BDDPM had a significant growth-inhibiting effect on Hela (IC_50_ = 17.63 μg/mL), RKO (IC_50_ = 11.37 μg/mL), HCT-116 (IC_50_ = 10.58 μg/mL), BEL-7402 (IC_50_ = 8.7 μg/mL) and U87 (IC_50_ = 23.69 μg/mL) cancer cell lines, and a minimal growth-inhibiting effect on the HUVEC (IC_50_ = 30.15 μg/mL) cell line. The results indicated that BDDPM had a significant growth-inhibiting effect on the cancer cell lines (Figure 1B). Among these cell lines, BEL-7402 cells were much more sensitive than the other cell lines. Based on this result, BEL-7402 cells were chosen for the subsequent experiments.

### 2.2. BDDPM Induces Morphological Changes and Apoptosis in BEL-7402 Cell

**Figure 2:**
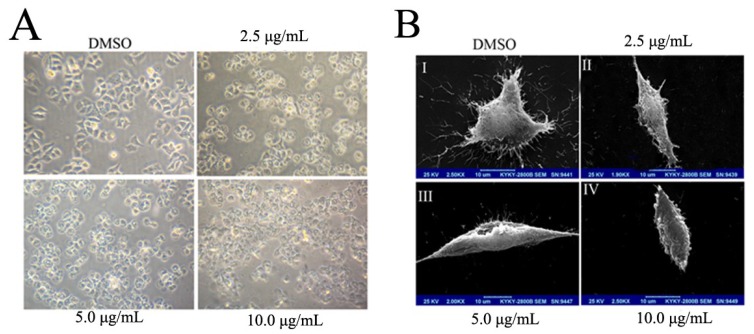
The morphological change of BEL-7402 cells upon treatment with BDDPM. (**A**) Cells were seeded in six-well plates (2 × 10^5^ cells/well) and allowed to adhere overnight. Then the cells were treated without or with 2.5, 5.0 and 10.0 μg/mL BDDPM for 12 h and subsequently observed under an inverted phase-contrast microscope (magnification, 100×); (**B**) For scanning electron microscopic observation, the BEL-7402 cells were grown on poly-L-lysine-coated cover slips for 24 h to allow firm attachment and treated with 2.5, 5.0, and 10.0 μg/mL BDDPM for 12 h. Cells were fixed on cover slips coated with gold and analyzed using the KYKY-2800B SEM. Cells were treated with DMSO were considered as negative controls (I). The remaining cells were treated with 2.5 μg/mL (II), 5.0 μg /mL (III) or 10.0 μg/mL (IV) of BDDPM. The BEL-7402 cells treated with DMSO showed a normal smooth surface with a lot of apophysis. In contrast, the cells treated with BDDPM became rounded, and the surface of the cell membrane was markedly disrupted (Scale bar = 10 μm).

Morphological changes were observed in cells under an invert microscope. The results showed that the BEL-7402 cells without treatment had the typical characters of human liver cancer cells, most of which were spindle-shaped with smooth edges and firmly attached to the surfaces of the cell culture dish (Figure 2A). However, the cells treated with BDDPM became spherical and detached from the plate surface, and most of them gradually became round with the increase in BDDPM concentration. The number of round cells increased in a dose-dependent manner. Cytoplasmic vacuoles could be detected in the rounded damaged cells under the high power microscope (Figure 2A).

The morphologic changes in the cell membrane were clearly visualized by scanning electronic microscopy (SEM). Remarkable alterations in the cell membrane of BEL-7402 cells were observed after BDDPM treatment. The architecture of untreated BEL-7402 cells displayed a typical polygonal shape (Figure 2B-I). However, the morphology of cells started to change after incubation with BDDPM. The cells were detached from the substratum, separated from each other, and became spindle-shaped (Figure 2B-II) after their exposure to 2.5 μg/mL of BDDPM. Membrane bulging and detachment from cytoplasmic inclusion were also observed in the cells with 5.0 μg/mL and 10.0 μg/mL BDDPM treatments (Figure 2B-III and Figure 2B-IV).

### 2.3. BDDPM Induces Apoptosis in BEL-7402 Cells

**Figure 3:**
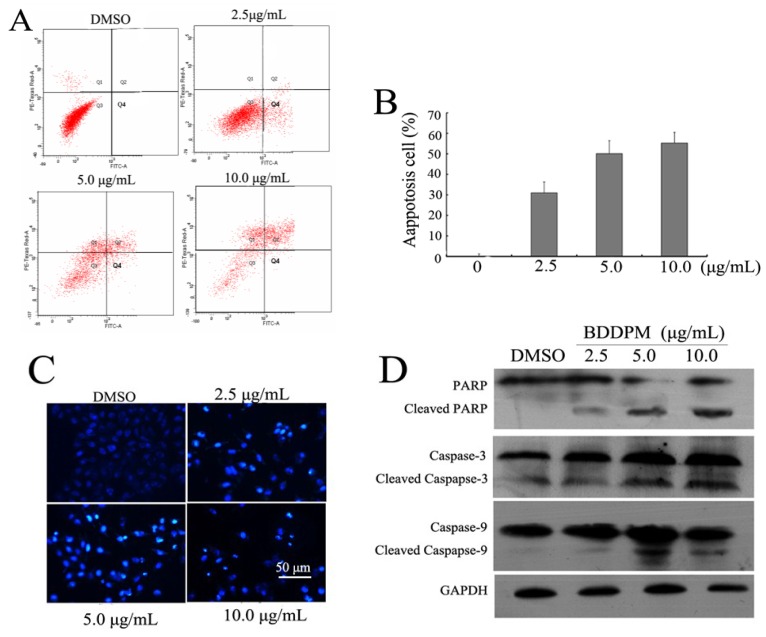
BDDPM induces BEL-7402 cell apoptosis. (**A**) Flow cytometric analysis of BDDPM-induced apoptosis in BEL-7402 cells. The percentage of Annexin V-FITC positive cells in the top (PI negative) and bottom (PI positive) right quadrant are indicated. Cells were treated with DMSO or treated with 2.5, 5.0, and 10.0 μg/mL of BDDPM for 48 h, respectively; (**B**) The histogram shows the percentage of early and late apoptosis and necrosis induced by BDDPM; (**C**) Analysis of apoptosis by staining with Hoechst 33342. The BEL-7402 cells were treated with DMSO or treated with 2.5, 5.0, or 10.0 μg/mL of BDDPM for 48 h. Cells were stained with Hoechst 33342 and observed under a fluorescence microscope; (Scale bar = 50 μm); (**D**) Immunoblot assays were applied to reveal the cleavages of Caspase 3, 9, and PARP in BEL-7402 treated with BDDPM. Glyceraldehyde 3-phosphate dehydrogenase (GAPDH) was used as loading control.

We further investigated the role of BDDPM in the apoptosis of BEL-7402 cells. Cells were treated with 0, 2.5, 5.0, and 10.0 μg/mL of BDDPM. After being cultured for 24–48 h, cells were collected, and AnnexinV-FITC and PI staining assays were performed to quantify the number of apoptotic cells. As shown in Figure 3A, BDDPM exposure resulted in an increase in the number of early apoptotic cells (AnnexinV-FITC-positive/PI-negative) in BEL-7402 in a dose-dependent manner. When treated with BDDPM at 2.5 and 10.0 μg/mL, the percentage of apoptotic cells was increased from 25.68% to 87.47%, respectively (Figure 3B). Subsequently, Hoechst 33342 staining was also performed to detect the apoptotic cells. Cell nuclear pyknosis, chromosome condensation and formation of apoptotic bodies were observed in BEL-7402 cells treated with BDDPM as detected by Hoechst 33342 staining. However, no apoptosis was found in cells treated with DMSO (Figure 3C). Cleavages of Caspase 3, 9, and poly ADP ribose polymerase (PARP) are important events for the activation of the intrinsic apoptotic pathway. Western blot analysis was performed to determine if BDDPM treatment resulted in cleavages of Caspases and PARP. The results showed that BDDPM promoted the cleavages of Caspase 3, 9, and PARP expression in a dose-dependent manner (Figure 3D). These results suggest that BDDPM induces cell death via the intrinsic apoptotic pathway.

### 2.4. BDDPM Affects the Migration and Invasion of BEL-7402 Cells

**Figure 4:**
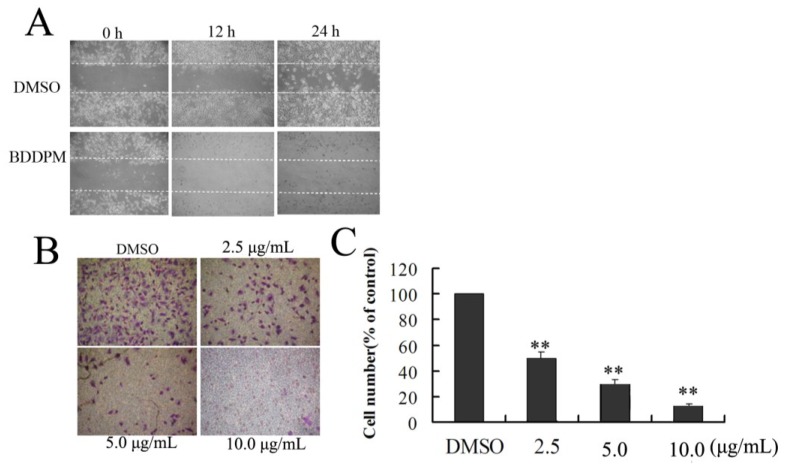
BDDPM inhibits BEL-7402 migration and invasion. (**A**) For the cell migration assay, BEL-7402 cells were treated with DMSO or 5.0 μg/mL BDDPM. After incubation for 12 h and 24 h, cell migration was analyzed using a scratch-wound assay; (**B**) For cell the invasion assay, BEL-7402 cells were treated with DMSO or with 2.5, 5.0 or 10.0 μg/mL BDDPM. After incubation for 24 h, non-invading cells on the upper surface of the membrane were removed and the invasive cells on the lower surface were stained with 0.1% crystal violet. The stained invasive cells were photographed under an inverted light microscope (100× magnifications); (**C**) Quantitative results of BEL-7402 cell invasion. Invasive cells were quantified by manual counting. The number represents the mean of six counting sights. Results are normalized to DMSO treated cells. All experiments were repeated more than three times. ** *p* < 0.01 *vs.* control.

Cell migration and invasion play crucial roles in tumor metastasis [11,18,20]. To further investigate the anti-metastatic effect of BDDPM, the ability of BEL-7402 cell migration was assessed by scratch-wound assay and transwell assays. The results from the scratch-wound assay showed that wound healing gradually reduced as the concentration of the BDDPM increased (Figure 4A).

We next investigated the anti-invasion activity of BDDPM on BEL-7402 cells using a transwell system. As shown in Figure 4B, treatment of BEL-7402 cells with BDDPM significantly inhibited the invasion of the cancer cells in a dose-dependent manner. When BEL-7402 was exposed to BDDPM at a concentration of 2.5, 5.0 and 10.0 μg/mL, the cell invasion to transwell was inhibited by 47.8%, 70.7%, and 86.2%, respectively (Figure 4B,C). These results suggested that BDDPM affected the ability of cell migration and invasion.

Both of the above findings indicated that BDDPM could significantly prevent BEL-7402 migration and invasion. Since inhibition of cell migration by BDDPM occurred before its inhibitory effect on cell proliferation was observed, the results suggest that BDDPM might indeed affect BEL-7402 cell migration and invasion, regardless of its effect on cell proliferation.

### 2.5. BDDPM Inhibits the Ability of BEL-7402 Cells to Adhere to ECM

**Figure 5:**
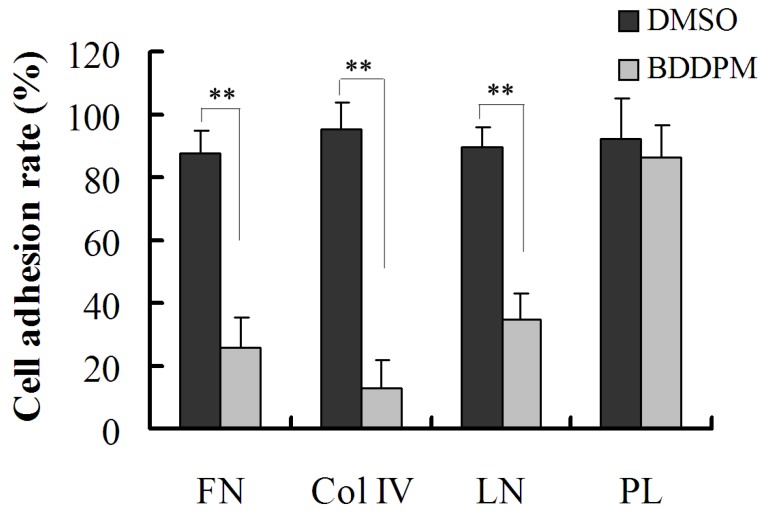
BDDPM affects Bel-7402 cell attachment to some extracellular matrix (ECM) proteins. Bel-7402 cells were suspended in serum-free medium containing 0.2% BSA without or with 5.0 μg/mL BDDPM and then seeded into pre-coated 96-well plates with 2.5 μg/mL fibronectin (FN), laminin (LN), poly-l-lysine (PL) or 5.0 μg/mL collagen IV (Col IV), respectively, and allowed to adhere for 1 h at 37 °C. After washing with PBS, the adhering cells were measured using an MTT assay. The adhesion rate of the treated cells was normalized to the control group. Data is shown as Mean ± SD from three independent experiments. ** *p* < 0.01 *vs.* control.

It is well known that some extracellular matrix (ECM) proteins, such as collagen IV, fibronectin (FN), and laminin (LN) play an important role in cell adhesion. To determine whether BDDPM affects some molecular events associated with cell attachment. The anti-adhesion effect of BDDPM on BEL-7402 cells was assessed by testing the adhesion ability of the cells to a cell matrix containing Col IV, FN, or LN. As shown in Figure 5, BDDPM remarkably reduced the adhesive ability of BEL-7402 cells to Col IV, FN or LN. Approximately 86.74% reduction in the number of cells adhering to Col IV gel was detected under the treatment of BDDPM (5.0 μg/mL), while exposure to the same concentration of BDDPM led to an adhesion of the BEL-7402 cells to the FN-containing matrix and a reduction of LN by 70.31% and 61.23%, respectively. However, BDDPM did not inhibit BEL-7402 cell adhesion to poly-l-lysine (*p* > 0.05), a non-ECM matrix. These results demonstrate that the treatment of BEL-7402 cells with BDDPM could inhibit the ability of these cells to adhere to ECM and result in cell detachment.

### 2.6. BDDPM Disrupts the Cytoskeleton and Changes the Morphology of BEL-7402

**Figure 6:**
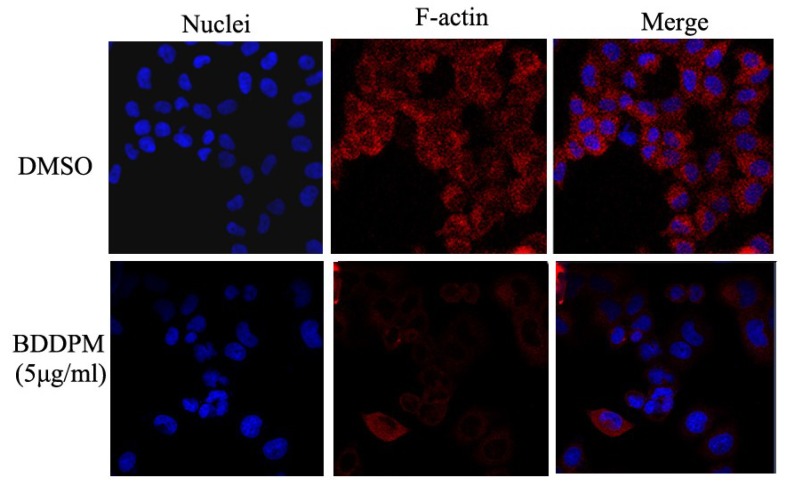
Effects of BDDPM on the BEL-7402 cell cytoskeleton. Human BEL-7402 cells were seeded onto cover slips coated with fibronectin and incubated over night prior to treatment (12 h, with or without 5.0 μg/mL BDDPM). Cells were then fixed and stained for F-actin (red) and the nuclei of the cells were stained using 4′,6-diamidino-2-phenylindole (DAPI) (blue); fluorescence images were viewed using a Zeiss confocal microscope (20×).

The effect of BDDPM on F-actin cytoskeleton organization was examined by immunofluorescence. As shown in Figure 6, BDDPM led to a dramatic disruption of the BEL-7402 cell cytoskeleton, producing a diffuse microtubule network and an increase in actin stress fibers and membrane blebbing. At the same time, cell morphology was significantly changed, with a rounded and retracted shape following exposure to BDDPM (Figure 6).

### 2.7. BDDPM Inhibits the Expression of β1-Integrin and FAK

**Figure 7:**
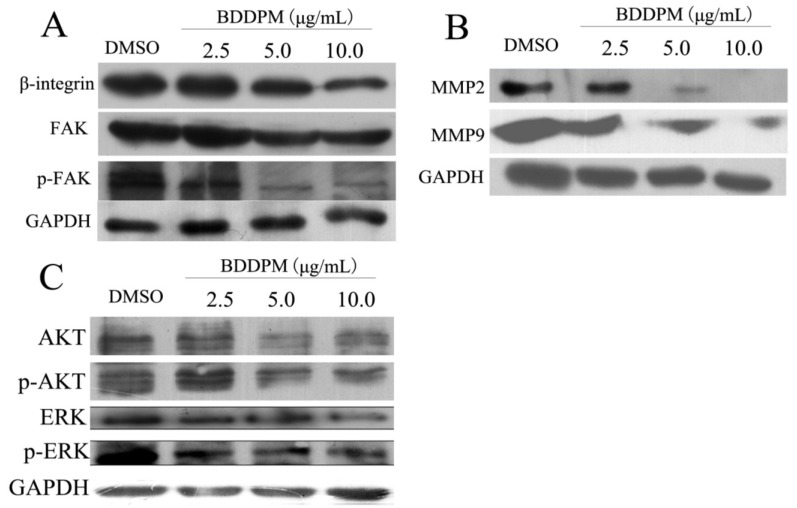
BDDPM disturbs β1-integrin/FAK signaling in BEL-7402 cells. BEL-7402 cells were treated with 2.5, 5.0 or 10.0 μg/mL BDDPM. After incubation for 24 h, cells were collected and the cell protein was isolated. (**A**) The expression level of β1-integrin, FAK, p-FAK were detected by Western blot; (**B**) The expression levels of MMP-2 and MMP-9 were measured by Western blot; (**C**) BDDPM inhibition of AKT and ERK phosphorylation. The anti-phospho-AKT antibody and anti-phospho-ERK antibody were used. The same membrane was stripped and re-probed using the anti-AKT, anti-ERK or anti-GAPDH antibody to detect total AKT and ERK levels. The cells treated with DMSO were used as negative control and the expression of GAPDH was used as a loading control.

To investigate the possible molecular mechanism underlying the effects of BDDPM on BEL-7402 cell behaviors, we performed flow cytometry and Western blot analysis to detect the expression of β1-integrin. Flow cytometrical analysis showed that, when the cells were treated with 5.0 μg/mL of BDDPM, the β1-integrin expression on the cell surface was significantly down-regulated in a dose-dependent manner (Figure 7A) compared to control cells. Accordingly, FAK, which is activated by β-integrin in normal and cancer cells, was significantly inhibited by BDDPM. Exposure to BDDMP resulted in the decrease in both total FAK protein and the activated FAK (phosphorylated FAK) expression levels (Figure 7A). Next, we detected the expression levels of MMP-2 and MMP-9, which are regulated by FAK and are critical for cancer cell invasion. The results revealed that treatment with BDDPM resulted in a significant decrease in the expression levels of MMP2 and MMP-9 in a dose-dependant manner (Figure 7B). PI3K/Akt and ERK are also in the downstream cascades of FAK signaling, and FAK phosphorylation of Akt/ERK suggests Akt and ERK activation. In the present study we examined the effects of BDDPM on Akt/ERK phosphorylation using antibodies recognizing phospho-serine 473 of Akt and phospho-Thr202/Tyr204 of ERK. The cells were incubated for 1 h in a medium containing 2.5–10.0 μg/mL of BDDPM. Drug treatment decreased the normalized levels of phospho-Akt and phospho-ERK in BEL-7402 cells (Figure 7C). This observation demonstrated that BDDPM inhibition of FAK kinase activity could decrease Akt and ERK activity. These results indicate that BDDPM inhibits proliferation, migration, and invasion of BEL-7402 cells by disturbing the β1-integrin/FAK signaling pathway.

## 3. Discussion

Tumor metastasis is a multistep process that involves tumor cell detachment from the primary tumor, adhesion to ECM or basement membrane, migration, invasion, angiogenesis, and metastatic tumor cell growth [20]. Tumor metastasis is also a major cause of death of cancer patients, and its blockage has been considered to benefit the survival of cancer patients [21]. Thus, it is crucial to identify new promising agents with anti-metastatic activity, which can disrupt or block metastasis. In this study, we found that the natural product BDDPM has antitumor activity on many types of cancer cells, and it has a potential to be developed as an anti-metastasis agent. Several lines of evidence suggest that BDDPM has anti-metastasis activity *in vitro*. Firstly, BDDPM has been found to induce cancer cell detachment and cause their apoptotic death. Secondly, BDDPM inhibits BEL-7402 cell adhesion to ECM and the major ECM proteins FN, LN, and Col IV. The promotion of detachment and inhibition of adhesion in BEL-7402 cancer cells mediated by BDDMP indicates that it disrupts the dynamic balance between attachment and detachment of cancer cells, whereas such dynamic balance regulates cell motility and is a fundamental premise for the metastasis of cancer cells [22,23]. It is assumed that BDDMP possesses anti-metastasis activity *in vitro*. Thirdly, wound a healing assay and a Boyden chamber assay revealed that BDDMP inhibites the migration and invasion of BEL-7402 cancer cells. Thus, BDDMP possesses anti-metastasis activity *in vitro* and could be developed as a novel anticancer agent.

Integrins are the major cell adhesion receptors that mediate cell adhesion to ECM proteins and influence diverse cellular functions crucial for the initiation, progression, and metastasis of solid tumors [24]. The importance of integrins for tumor progression has made them an appealing target for cancer therapy. In recent years, various integrin antagonists or inhibitors have been developed. Cilengitide, an inhibitor of both αvβ3 and αvβ5 integrins, has been tested in Phase II trials in patients with lung and prostate cancer [25], and Phase II and Phase III trials are currently underway for glioblastoma treatment [26,27,28]. Volociximab, a function-blocking antibody against σ5β1 integrin, is currently undergoing Phase II clinical trials for solid tumors [29]. In the present study, we also found that BDDMP had a direct effect on the expression of β1-integrin (Figure 6). The β1-integrin is a candidate target well known for mediating cell-ECM interactions. Recent studies have shown that its aberrant expression is implicated in cancer progression and the resistance to cytotoxic therapy [30]. With the down-regulation of β1-integrin by siRNA or miR-134, the cell adhesion and invasiveness were highly inhibited [31,32]. Our findings indicate that BDDPM could be developed into a novel inhibitor of β1-integrin. However, further studies are needed to verify BDDPM-induced down-regulation of β1-integrin in BEL-7402 cancer cells, as well as in other cancer cell lines. A study is in progress in our laboratory to investigate if the treatment of cancer cells with BDDPM affects the expression of other types of integrins.

FAK activation and phosphorylation stimulated by integrins is critical for anchorage-independent growth of cancer cells. Several studies have revealed that increased FAK expression is correlated with enhanced tumor malignancy and poor prognosis [33]. Recent studies that employed RNAi to inhibit FAK expression in carcinoma cells have been yielding insights into FAK’s role for tumor growth and spreading, and have also revealed that FAK expression and activity are essential for cell invasiveness [12]. FAK is required for cell transformation and invasion [13,23,34], which has become an attractive target for drug discovery. To determine the effects of BDDPM-inhibited FAK on downstream signaling and cell phenotypes, the important downstream genes including AKT/ERK were also investigated in the present study, and we found that BDDPM inhibition of FAK activity in BDDPM cells resulted in a lower AKT/ERK phosphorylation/activity, which is correlated with decreased survival and increased apoptosis (cleaved PARP and Caspases increased). Besides, BDDPM inhibits cell migration by downregulation of the MMP-2 and MMP-9. All the results indicate that BDDPM targets FAK kinase activity and inhibits FAK-related AKT/ERK activation, which impacts cell viability, and decreases anchorage-independent growth and motility. In summary, the present study found that BDDPM could disturb the integrin-FAK signal transduction and that the FAK expression level was decreased by BDDPM treatment, indicating that BDDPM has a potential to be developed as an intergrin-FAK inhibitor.

## 4. Experimental Section

### 4.1. Materials

BDDPM was first isolated from *Rhodomelaceae confervoides*, and was subsequently synthesized by our lab (purity, 98%) [9]. The Dulbecco’s modified Eagle’s medium (DMEM), fetal bovine serum (FBS), and other cell culture reagents were purchased from Invitrogen (Carlsbad, CA, USA). The high concentration ECM gel was purchased from BD Biosciences (BD Biosciences, Bedford, MA, USA).

### 4.2. MTT Assay

MTT assay was performed as previously reported [35]. Briefly, cells were seeded in 96-well plates (5 × 10^3^ cells/well) and allowed to adhere overnight. After the cells were treated with specific doses of samples for 24 h at 37 °C, a MTT solution (30 μL, 5 mg/mL) was added into each well and the plates were incubated for 4 h before the MTT-containing solution was removed and replaced with 150 μL of DMSO. The absorbance at 490 nm was then determined with an ELx808 microplate reader (BioTek, Winooski, VT, USA). The viability rate of the treated cells was calculated by the formula: cell viability rate (%) = [(A490 sample)/(A490 control)] × 100%. The IC50 value was deduced from the MTT dose-response curves of cell viability against drug concentration.

### 4.3. Cell Morphological Observation

Morphologic alterations in BEL-7402 cells after BDDPM treatment were observed and photographed under an inverted microscope (Carl Zeiss, Oberkochen, Germany) or scanning electron microscope (SEM). For inverted microscope observation, cells in logarithmic phase were suspended in 1640 medium with 10% FBS and seeded into 24-well plates (50,000 cells/well). After incubation for 24 h, cells were treated with BDDPM (0, 2.5, 5.0, 10.0 μg/mL) and cultured for 12 h. Cell morphological changes were observed and photographed under an inverted microscope (Carl Zeiss, Oberkochen, Germany). For scanning electron microscope assay, the cells were grown on poly-l-lysine-coated coverslips in six-well plates for 24 h to allow firm attachment. Cells were then treated with 2.5, 5.0, 10.0 μg/mL BDDPM and incubated for 12 h. The medium containing BDDPM was removed, and the cells were subsequently fixed in 0.25% glutaraldehyde. After fixation overnight at 4 °C, the coverslips were dehydrated with ethanol and dried in a critical point dryer. Cells on cover slips were coated with gold and analyzed with the S-3400N SEM (Hitachi, Lexington, KY, USA).

### 4.4. Hoechst33342/Propidium Iodide (PI) Dual Staining Assays

The apoptotic cells were stained using Hoechst33342/PI double staining as we described previously [35]. The BEL-7402 cells were seeded in six-well plates (2 × 10^5^ cells/well) and treated with certain concentrations of BDDPM (2.5, 5.0, 10.0 μg/mL). After incubation for 24 h, the cells were collected and washed with PBS. Cells were stained with Hoechst33342 and PI using the dual staining kit (Beyond, Beijing, China). Then the cells were spread on slides and observed under the fluorescence microscope (Carl Zeiss, Oberkochen, Germany).

### 4.5. Apoptosis Assay

Cell apoptosis was detected by Annexin V-FITC assay. Apoptotic cell death was quantified by flow cytometry with Annexin V-FITC and propidium iodide (PI) staining. Annexin V-FITC apoptosis detection kit (Invitrogen, Carlsbad, CA, USA) was used according to the manufacturer’s instructions. Briefly, both attached and floating cells were collected and resuspended in binding buffer before adding the Annexin V-FITC antibody and PI. Stained cells were analyzed by flow cytometry (Beckman Coulter,Brea, CA, USA).

### 4.6. Cell Adhesion Assay

The cell adhesion assay was performed as described previously [19]. Briefly, Bel-7402 cells were suspended in serum-free medium containing 0.2% BSA without or with 5 μg/mL BDDPM and then seeded in precoated 96-well plates with 2.5 μg/mL fibronectin (FN) and laminin (LN), poly-l-lysine (PL) or 5 μg/mL collagen IV (Col IV), respectively, and allowed to adhere for 1 h at 37 °C. After washing with PBS, the adherent cells were measured using an MTT assay. The adherent rate of the treated cells was normalized to the control group.

### 4.7. Cell Migration Assay

The migration ability of BEL-7402 cells was assessed using a modified version of a previously described protocol [34] with a transwell system (Corning, Tewksbury, MA, USA). Cells (10,000 cells/well) were added to the upper chamber of the transwell culture plates, in the presence and absence of certain concentrations of BDDPM (0.0–10.0 μg/mL). The lower chamber was filled with 500 μL F-12K medium containing 10% FBS as a chemo-attractant. After incubation for 12 h, non-migrating cells on the upper surface of the membrane were scrubbed gently with a cotton-tipped swab. The migratory cells on the lower surface of the membrane were fixed with 95% methanol, stained with 0.1% crystal violet (Sigma-Aldrich, St. Louis, MO, USA), counted with inverted microscope and quantified by manual counting in three randomly selected areas.

### 4.8. Cell Invasion Assay

The effect of BDDPM on BL-7402 cell invasion was measured by a transwell system with a diameter of 6.5 mm and a pore size of 8 μm. ECM gel was applied to the top side of the filter to form a thin gel layer. As described above for the cell migration assay, cells that penetrated to the lower chamber were fixed and stained. The stained invasive cells were photographed under an inverted light microscope and quantified by manual counting in three randomly selected areas.

### 4.9. Cytoskeleton Immunofluorescence

BEL-7402 cells were seeded on ploy lysine-coated chamber slides before exposure to BDDPM (10 μg/mL) for 4 h. The cells were then harvested and fixed with 4% paraformaldehyde, permeabilized with 0.1% Triton X-100, and blocked with 5% BSA solution. The microtubules were then labeled with mouse monoclonal anti-F-actin antibody (1:500, Santa Cruz, Dallas, TX, USA), followed by incubated with the second antibody (Alexa Fluor549-anti-Rabbit IgG, 1:1000, Life science, St. Louis, MO, USA), after that the cell nuclei were stained with DAPI (5 mg/mL) for five minutes and the Fluorescence images were obtained by using confocal microscope (Zeiss, Oberkochen, Germany).

### 4.10. Western Blot Analysis

Cells were lysed in RIPA buffer (Solaibo, Beijing, China). Proteins were separated by a 10% polyacrylamide gel and transferred to a methanol-activated PVDF membrane (GE Healthcare, Little Chalfont, Buckinghamshire, UK). The membrane was blocked in blocking solution (5% nonfat dry milk powder) for 2 h at room temperature and subsequently probed with primary antibodies; including ILK, β1- integrin, PARP, Caspase-3, Caspase-9, total/phospho FAK, total/phospho-Akt, total/phospho-ERK, (used at a 1/1000 dilution, Santa Cruz Biotechnology, Santa Cruz, CA, USA), MMP2, MMP9 (used at a 1/1000 dilution, AbClonal, USA) Or GAPDH (used at a 1/5000 dilution, AbClonal, Cambridge, MA, USA). After three 10 min washes with 0.1% Tween-20 in PBS buffer, membranes were incubated with rabbit anti mouse or goat anti rabbit HRP-conjugated secondary antibody (Santa Cruz) for 1 h. After an additional three 10 min washes with 0.1% Tween-20 in PBS buffer, the chemiluminescence method was employed to detect the signals using Super Signal West Dura (Thermo Scientific, Waltham, MA, USA) and protein bands were visualized by autoradiography. 

### 4.11. Statistical Analysis

Statistical significance of the data was analyzed by the two-tail Student’s *t*-test with a minimum significance level set at *p* < 0.05 (marked as * *p* < 0.05 and ** *p* < 0.01).

## 5. Conclusions

This study suggests that the natural marine bromphenol compound Bis (2,3-dibromo-4,5-dihydroxy-phenyl)-methane (BDDPM) induces cancer cell detachment and causes their apoptotic death, and it has a potential to be developed as a novel anticancer therapeutic agent due to its anti-metastatic activity. BDDPM inhibits cell migration and invasion by targeting β1-integrin/FAK signaling.

## References

[1] Liu M., Wang G., Xiao L., Xu X., Liu X., Xu P., Lin X. (2014). Bis(2,3-dibromo-4,5-dihydroxybenzyl) ether, a marine algae derived bromophenol, inhibits the growth of botrytis cinerea and interacts with DNA molecules. Mar. Drugs.

[2] Liu M., Zhang W., Wei J., Qiu L., Lin X. (2012). Marine bromophenol bis(2,3-dibromo-4,5-dihydroxybenzyl) ether, induces mitochondrial apoptosis in k562 cells and inhibits topoisomerase I *in vitro*. Toxicol. Lett..

[3] Ma M., Zhao J., Wang S., Li S., Yang Y., Shi J., Fan X., He L. (2006). Bromophenols coupled with methyl gamma-ureidobutyrate and bromophenol sulfates from the red alga rhodomela confervoides. J. Nat. Prod..

[4] Wang B.G., Gloer J.B., Ji N.Y., Zhao J.C. (2013). Halogenated organic molecules of rhodomelaceae origin: Chemistry and biology. Chem. Rev..

[5] Liu M., Zhang W., Wei J., Lin X. (2011). Synthesis and alpha-glucosidase inhibitory mechanisms of bis(2,3-dibromo-4,5-dihydroxybenzyl) ether, a potential marine bromophenol alpha-glucosidase inhibitor. Mar. Drugs.

[6] Pereira R., Benedetti R., Perez-Rodriguez S., Nebbioso A., Garcia-Rodriguez J., Carafa V., Stuhldreier M., Conte M., Rodriguez-Barrios F., Stunnenberg H.G. (2012). Indole-derived psammaplin a analogues as epigenetic modulators with multiple inhibitory activities. J. Med. Chem..

[7] Shi D.Y., Li J., Guo S.J., Su H., Fan X. (2009). The antitumor effect of bromophenol derivatives *in vitro* and *Leathesia nana* extract *in vivo*. Chin. J. Oceanol. Limn..

[8] Oh K.B., Lee J.H., Lee J.W., Yoon K.M., Chung S.C., Jeon H.B., Shin J., Lee H.S. (2009). Synthesis and antimicrobial activities of halogenated bis(hydroxyphenyl)methanes. Bioorg. Med. Chem. Lett..

[9] Li J., Guo S.J., Su H., Han L.J., Shi D.Y. (2008). Total synthesis of bis-(2,3-dibromo-4,5-dihydroxyphenyl)-methane as potent PTP1B inhibitor. Chin. Chem. Lett..

[10] Bauvois B. (2012). New facets of matrix metalloproteinases mmp-2 and mmp-9 as cell surface transducers: Outside-in signaling and relationship to tumor progression. Bba-Rev. Cancer.

[11] Ma W.L., Jeng L.B., Lai H.C., Liao P.Y., Chang C. (2014). Androgen receptor enhances cell adhesion and decreases cell migration via modulating beta 1-integrin-Akt signaling in hepatocellular carcinoma cells. Cancer Lett..

[12] Mitra S.K., Schlaepfer D.D. (2006). Integrin-regulated FAK-Src signaling in normal and cancer cells. Curr. Opin. Cell Biol..

[13] Yao W.L., Ko B.S., Liu T.A., Liang S.M., Liu C.C., Lu Y.J., Tzean S.S., Shen T.L., Liou J.Y. (2014). Cordycepin suppresses integrin/FAK signaling and epithelial-mesenchymal transition in hepatocellular carcinoma. Anti-Cancer Agent Me.

[14] Eke I., Deuse Y., Hehlgans S., Gurtner K., Krause M., Baumann M., Shevchenko A., Sandfort V., Cordes N. (2012). Beta(1) integrin/FAK/cortactin signaling is essential for human head and neck cancer resistance to radiotherapy. J. Clin. Invest..

[15] Saleem S., Li J.M., Yee S.P., Fellows G.F., Goodyer C.G., Wang R.N. (2009). Beta 1 integrin/FAK/Erk signalling pathway is essential for human fetal islet cell differentiation and survival. J. Pathol..

[16] Bouchard V., Harnois C., Demers M.J., Thibodeau S., Laquerre V., Gauthier R., Vezina A., Noel D., Fujita N., Tsuruo T. (2008). Beta 1 integrin/FAK/Src signaling in intestinal epithelial crypt cell survival: Integration of complex regulatory mechanisms. Apoptosis.

[17] Han J.W., Lee H.J., Bae G.U., Kang J.S. (2011). Promyogenic function of integrin/FAK signaling is mediated by Cdo, Cdc42 and MyoD. Cell Signal..

[18] Huttenlocher A., Sahai E. (2014). Editorial overview: Cell adhesion and migration. Curr. Opin. Cell Biol..

[19] Wang F.X., Wu N., Wei J.T., Liu J., Zhao J., Ji A.G., Lin X.K. (2013). A novel protein from eupolyphaga sinensis inhibits adhesion, migration, and invasion of human lung cancer A549 cells. Biochem. Cell Biol..

[20] Woodhouse E.C., Chuaqui R.F., Liotta L.A. (1997). General mechanisms of metastasis. Cancer.

[21] Lee Y.S., Nam K.T., Lee S., Yu D., Choi G., Shin S.K., Lee Y.C. (2012). Regulation of epithelial mesenchymal transition through protein kinase CK2 in helicobacter pylori infected gastric cancer cells. Gastroenterology.

[22] Lauffenburger D.A. (1996). Cell motility. Making connections count. Nature.

[23] Lauffenburger D.A., Horwitz A.F. (1996). Cell migration: A physically integrated molecular process. Cell.

[24] Desgrosellier J.S., Cheresh D.A. (2010). Integrins in cancer: Biological implications and therapeutic opportunities. Nat. Rev. Cancer.

[25] Beekman K.W., Colevas A.D., Cooney K., Dipaola R., Dunn R.L., Gross M., Keller E.T., Pienta K.J., Ryan C.J., Smith D. (2006). Phase II evaluations of cilengitide in asymptomatic patients with androgen-independent prostate cancer: Scientific rationale and study design. Clin. Genitourin Cancer.

[26] Manegold C., Vansteenkiste J., Cardenal F., Schuette W., Woll P.J., Ulsperger E., Kerber A., Eckmayr J., von Pawel J. (2013). Randomized phase II study of three doses of the integrin inhibitor cilengitide *vs.* docetaxel as second-line treatment for patients with advanced non-small-cell lung cancer. Invest. New Drugs.

[27] Kim K.B., Prieto V., Joseph R.W., Diwan A.H., Gallick G.E., Papadopoulos N.E., Bedikian A.Y., Camacho L.H., Hwu P., Ng C.S. (2012). A randomized phase II study of cilengitide (EMD 121974) in patients with metastatic melanoma. Melanoma Res..

[28] Reardon D.A., Fink K.L., Mikkelsen T., Cloughesy T.F., O’Neill A., Plotkin S., Glantz M., Ravin P., Raizer J.J., Rich K.M. (2008). Randomized phase II study of cilengitide, an integrin-targeting arginine-glycine-aspartic acid peptide, in recurrent glioblastoma multiforme. J. Clin. Oncol..

[29] Besse B., Tsao L.C., Chao D.T., Fang Y., Soria J.C., Almokadem S., Belani C.P. (2013). Phase IB safety and pharmacokinetic study of volociximab, an anti-alpha5beta1 integrin antibody, in combination with carboplatin and paclitaxel in advanced non-small-cell lung cancer. Ann. Oncol..

[30] Park C.C., Zhang H., Pallavicini M., Gray J.W., Baehner F., Park C.J., Bissell M.J. (2006). Beta1 integrin inhibitory antibody induces apoptosis of breast cancer cells, inhibits growth, and distinguishes malignant from normal phenotype in three dimensional cultures and *in vivo*. Cancer Res..

[31] Zha R., Guo W., Zhang Z., Qiu Z., Wang Q., Ding J., Huang S., Chen T., Gu J., Yao M. (2014). Genome-wide screening identified that miR-134 acts as a metastasis suppressor by targeting integrin beta1 in hepatocellular carcinoma. PLoS One.

[32] Speicher T., Siegenthaler B., Bogorad R.L., Ruppert R., Petzold T., Padrissa-Altes S., Bachofner M., Anderson D.G., Koteliansky V., Fassler R. (2014). Knockdown and knockout of beta1-integrin in hepatocytes impairs liver regeneration through inhibition of growth factor signalling. Nat. Commun..

[33] Hsia D.A., Mitra S.K., Hauck C.R., Streblow D.N., Nelson J.A., Ilic D., Huang S., Li E., Nemerow G.R., Leng J. (2003). Differential regulation of cell motility and invasion by FAK. J. Cell Biol..

[34] Wu X., Gan B., Yoo Y., Guan J.L. (2005). Fak-mediated src phosphorylation of endophilin A2 inhibits endocytosis of MT1-MMP and promotes ECM degradation. Dev. Cell.

[35] Wu N., Lin X., Zhao X., Zheng L., Xiao L., Liu J., Ge L., Cao S. (2013). miR-125b acts as an oncogene in glioblastoma cells and inhibits cell apoptosis through p53 and p38MAPK-independent pathways. Br. J. Cancer.

